# Galectin-3 deficiency exacerbates hyperglycemia and the endothelial response to diabetes

**DOI:** 10.1186/s12933-015-0230-3

**Published:** 2015-06-06

**Authors:** April L. Darrow, Ralph V. Shohet

**Affiliations:** Center for Cardiovascular Research and Department of Medicine, University of Hawaii John A. Burns School of Medicine, Honolulu, HI USA; Department of Cell and Molecular Biology, University of Hawaii John A. Burns School of Medicine, 651 Ilalo Street, Honolulu, HI 96813 USA

**Keywords:** Endothelial dysfunction, Vasculopathy, Microarray analysis, GLUT4, Type II diabetes

## Abstract

**Background:**

Diabetes promotes maladaptive changes in the endothelium that lead to its dysfunction and contribute to the vascular pathology of diabetes. We have previously reported the up-regulation of galectin-3, a β-galactoside-binding lectin, in the endothelium and sera of diabetic mice, implicating this molecule in diabetic vasculopathy and suggesting its potential as a biomarker of the disease. Therefore, we sought to assess the role of galectin-3 in the vascular pathology of diabetes.

**Methods:**

Galectin-3 knockout mice (KO) and wild-type mice (WT) were fed either a high-fat diet (HFD) (60 % fat calories) to produce insulin resistant diabetes, or standard chow (12 % fat calories), and their metabolic and endothelial responses were measured. After 8 weeks, the aortic and skeletal muscle endothelia were isolated by fluorescence sorting of CD105^+^/CD45^−^ cells and comprehensive transcriptional analyses were performed. Transcripts differentially dysregulated by HFD in KO endothelium compared to WT were confirmed by semi-quantitative RT-PCR, and protein expression was determined by immunofluorescence of aortic and muscle tissue. Ingenuity® Pathway Analysis was used to identify pathways up-regulated by HFD in the KO, such as the coagulation cascade, and measurements of blood clotting activity were performed to confirm these results.

**Results:**

KO mice demonstrate greater hyperglycemia and impaired glucose tolerance but lower insulin levels on HFD compared to WT. KO mice demonstrate a more robust transcriptional response to HFD in the vascular endothelium compared to WT. Transcripts dysregulated in the KO endothelium after HFD are involved in glucose uptake and insulin signaling, vasoregulation, coagulation, and atherogenesis. One of the most down-regulated transcripts in the endothelium of the KO after HFD was the glucose transporter, *Glut4/Slc2a4.* GLUT4 immunofluorescence confirmed lower protein abundance in the endothelium and muscle of the HFD-fed KO. Prothrombin time was decreased in the diabetic KO indicating increased coagulation activity.

**Conclusions:**

Galectin-3 deficiency leads to exacerbated metabolic derangement and endothelial dysfunction. The impaired tissue uptake of glucose in KO mice can be attributed to the reduced expression of GLUT4. Enhanced coagulation activity in the diabetic KO suggests a protective role for galectin-3 against thrombosis. These studies demonstrate that galectin-3 deficiency contributes both to the pathogenesis of diabetes and the associated vasculopathy.

**Electronic supplementary material:**

The online version of this article (doi:10.1186/s12933-015-0230-3) contains supplementary material, which is available to authorized users.

## Background

Diabetes promotes maladaptive vascular responses in numerous tissues, including changes in the endothelium that contribute to insulin resistance and ultimately, the vascular pathology of diabetes. Endothelial damage or dysfunction is the primary cause of the macrovascular and microvascular complications associated with diabetes. Previously, we have developed methods for the rapid isolation of highly purified endothelial cells from mice exposed to models of diabetes [1, 2]. Transcriptional analysis of these diabetic endothelial cells revealed increased abundance of galectin-3 mRNA and protein in the aortic and muscle endothelium. We also found a correlation between serum galectin-3 levels and insulin resistance, implicating this molecule as a potential biomarker of diabetic vasculopathy [1]. Human studies have confirmed an elevation of systemic galectin-3 in type II diabetic patients compared to healthy individuals [3]. A recent clinical study has shown that galectin-3 levels correlate with plasma glucose, C-reactive protein, and degree of insulin resistance, suggesting its potential as a biomarker for predicting diabetes and prediabetes [4]. Higher galectin-3 levels have also been associated with increased risk of vascular complications [5]. In light of our preliminary findings, we suspected that galectin-3 is involved in the endothelial dysfunction that leads to the vascular complications of type II diabetes. Thus, we sought to determine the role of galectin-3 in the vascular pathology and metabolic derangement observed in type II diabetes.

In recent years, the importance of this molecule in disease pathology and its potential as a therapeutic target has become increasingly evident. Galectin-3 has been studied for putative roles in heart failure, fibrosis, cancer, angiogenesis, and atherosclerosis. Human studies show that subjects with elevated galectin-3 levels have increased risk for developing heart failure, and blood testing for galectin-3 is now being used to monitor heart failure [6–8]. Perhaps similar testing could be used to predict vascular complications in diabetic patients.

Intracellular LGALS3 is involved in pre-mRNA splicing and has been implicated in the regulation of gene transcription by stabilizing transcription factor binding [9, 10]. Extracellular LGALS3 interacts with components of the extracellular matrix as well as cell-surface carbohydrates to influence cell adhesion and migration. LGALS3 is up-regulated on the endothelial cell surface during adherence and migration of polymorphonuclear leukocytes *in vitro* [11] and may also be involved in mediating NG2 proteoglycan-induced motility leading to the recruitment of endothelial cells to sites of neovascularization [12]. Galectin-3 is also a component of the advanced glycation endproduct (AGE)–receptor complex expressed on the surface of endothelial cells, which is linked to the binding and removal of AGEs from the circulation [13]. CHO cells overexpressing LGALS3 specifically bind AGE-BSA and modified LDLs, leading to their endocytosis [14]. Galectin-3 deficient mice have been shown to display increased glomerular accumulation of AGEs in a model of type I diabetes and increased ox-LDL and lipoprotein products when fed an atherogenic diet [15, 16].

The galectin-3 knockout has been used to study the role of galectin-3 in murine models of type I diabetes induced by streptozotocin, where both pro-diabetogenic and protective roles have been reported [15, 17]. Galectin-3 protein expression increases in human atherosclerotic lesions as well as in the aortae of experimental animal models of diabetes [18, 19]. Galectin-3 deletion or inhibition on the *ApoE (−/−)* background has been shown to reduce atherosclerotic lesions and plaques [18, 20], while galectin-3 knockout mice fed an atherogenic diet for 8 months showed increased lesion area and length compared to wild-type mice [16]. These studies suggest an important role for galectin-3 in vascular complications. However, the conflicting reports regarding its role in diabetes and its associated vasculopathy emphasize the need for further evaluation.

The goal of our study was to identify the role of this multifunctional lectin in the development of endothelial dysfunction induced by type II diabetes. To do this, we examine the metabolic changes and the endothelial transcriptional response to a high-fat diet in wild-type and *Lgals3*-deficient mice. These studies define the role of galectin-3 in endothelial dysfunction and insulin resistance and help to elucidate the pathogenesis of diabetic vasculopathy.

## Methods

### Animals and diet

Mice homozygous for a targeted deletion within the galectin-3 gene (KO) [B6.Cg-*Lgals3*^*tm1Poi*^/*J*, stock no. 006338 Jackson Laboratories (Bar Harbor, ME)] and wild-type C57BL/6 J (WT) (stock no. 000664) were bred for these experiments. The galectin-3 knockout was generated by a 3.7 kb deletion of exons 2–4, including the initiating codon in exon 2 as previously described [21]. Genetic deletion of galectin-3 was confirmed at the DNA level by gel electrophoresis and also at the RNA and protein level by real-time PCR and Western blotting (Additional file 1).

Beginning at 8 weeks of age, male KO and WT mice were allowed to feed ad libitum on a high-fat diet (HFD) containing 60 % fat calories (BioServ, Frenchtown, NJ, cat. no. S3282) or a normal chow diet containing 12 % fat calories (LabDiet, St. Louis, MO, cat. no. 5001) for a period of 8 weeks. All procedures were approved by the Institutional Animal Care and Use Committee of the University of Hawaii.

### Metabolic parameters

Blood glucose and serum insulin levels were measured before the start of the diet and after 2, 4, 6, and 8 weeks on their respective diets. Animals were fasted overnight, and glucometry of tail blood was performed using an OneTouch Ultra (Lifescan, Milpitas, CA). Two-hundred μL of blood was collected from the tail vein, and insulin levels in the separated serum were determined by ELISA (Mercodia, Sweden). A glucose tolerance test (GTT) was performed after 5 weeks on the diet. Glucose (1 mg/g of body weight) was administered i.p. following an overnight fast, and glucometry of the tail blood was performed prior to glucose injection and every 20 minutes afterwards for 2 hrs. The homeostatic model assessment of insulin resistance (HOMA-IR) was calculated using the following formula: [fasting blood glucose (mg/dL) × fasting insulin (μIU/mL)] / 405 [22], where 1 mg insulin = 26 IU [23].

Advanced glycation end products (AGEs) in the serum were measured after 8 weeks of diet. Blood was collected by cardiac puncture and serum was separated and diluted to obtain a protein concentration of 5 mg/mL. AGEs were quantified by ELISA (Wuhan EIAAB Science, China, cat.no.E0263m) and normalized to total protein.

### Measurement of AKT phosphorylation

Following 8 weeks on the diet, chow and high-fat fed mice of each strain received an i.p. injection of insulin (0.06 U/g body weight in 300 μL of sterile saline); 1–2 animals of each group received vehicle (normal saline). Fifteen minutes after insulin challenge, animals were sacrificed and thoracic aorta and skeletal muscle were harvested and homogenized as previously described [1]. Abundance of phosphorylated AKT and total AKT were determined by Western Blotting. Briefly, denatured aorta or muscle protein was separated on 12 % Bis-Tris polyacrylamide gels under reducing conditions. Following transfer and blocking, membranes were incubated overnight in rabbit anti-mouse phospho-AKT antibody (serine 473) (Cell Signaling, Beverly, MA, cat.no.9271) at 4 °C and visualized with an Alexa Fluor®568-conjugated secondary antibody (Invitrogen, Carlsbad, CA) on a Typhoon® phosphorimager (Amersham). Blots were then stripped and reprobed for total AKT (Cell Signaling, cat.no.9272). Band intensity was quantified by densitometry using ImageJ [24], and phosphorylated AKT was normalized to total AKT levels.

### Endothelial cell isolation

Following 8 weeks on the diet regimen, animals were sacrificed by CO_2_ asphyxiation. In each experiment, for each diet, pooled cells from 3 KO and 3 WT mice were collected for each tissue. Aortae from the aortic root to the iliac bifurcation were dissected. Leg muscles consisting of the plantaris, gastrocnemius, and biceps femoris (which are readily dissected as a single group) were excised. Tissues were placed into ice cold PBS and freed of adherent fat. The aortic and skeletal muscle tissues from 3 animals were each pooled, minced into 1 mm fragments, and dispersed as previously described [25]. Suspensions of collagenolytically separated cells were incubated with anti-mouse CD16/32 (1:500) for 5 min and then with phycoerythrin-conjugated anti-mouse CD45 (1:800) and anti-mouse CD105 eFluor®450 (1:20) for 25 minutes on ice (eBiosciences, cat.nos.14-0161, 17–0451, and 48–1051). Immediately before sorting, 5 μl of Dead Cell Discriminator (Life Technologies, Carlsbad, CA) was added per 100 μl of cell suspension. Roughly 10,000 live endothelial cells positive for CD105 staining and negative for CD45 were isolated with a FACSAria (Becton Dickinson, Franklin Lakes, NJ) directly into Trizol (Invitrogen, Carlsbad, CA). RNA was extracted and purified with RNeasy columns (Qiagen, Valencia, CA) and then amplified using an Ambion® Amino Allyl MessageAmp kit (Life Technologies, Carlsbad, CA) according to the manufacturer’s protocol to produce approximately 100 μg of amino-allyl modified cRNA.

### Transcriptional analysis

Microarray analyses were performed to determine the transcriptional responses in aortic and skeletal muscle endothelium from KO vs. WT mice exposed to either high-fat diet or control diet for 8 weeks. Amino-allyl modified cRNA was labeled with Cy3 CyDye™ Post-Labeling Reactive Dye Pack (GE Healthcare, Waukesha, WI) according to the manufacturer’s instructions. Following purification, 1.6 μg of Cy3 dye-labeled cRNA, as measured by NanoDrop 2000c spectrophotometer (Thermo Scientific, Bellerica, MA), were combined and fragmented. Biological triplicate experiments were performed for both tissues and both diets for each strain. Samples were hybridized overnight to glass slides spotted with the Whole Mouse Genome Array Kit from Agilent (Santa Clara, CA, cat. no. G4122F), which includes 44,000 murine oligonucleotides representing all known genes and transcripts of the mouse genome along with positive controls. The set of 3’ biased, 60-mer oligos are synthesized *in situ* using Agilent SurePrint technology. Annealed fluorescent labels were quantified with Agilent dual channel scanner, and data analysis subsequently performed with GeneSpring® and Acuity® software. For both the KO and WT strains, transcripts exhibiting up-regulation (a log_2_ [fold change] >1) or down-regulation (log_2_ [fold change] < −1) by HFD vs. chow diet that were found consistently dysregulated in all 3 biologically replicate experiments were tabulated for each tissue. This comparison represents the endothelial response to diabetes in either strain.

### Pathway analysis

The gene expression data was further interrogated using QIAGEN’s Ingenuity® Pathway Analysis (IPA®, QIAGEN Redwood City, www.qiagen.com/ingenuity). Datasets consisting of all transcripts differentially expressed >0.75 log_2_ [fold change] by high-fat feeding in the WT and KO strains were uploaded into IPA®. Over-represented Biological Functions in the aortic endothelium of each strain after HFD were identified based on weighted gene co-expression. IPA was also performed on differentially regulated transcripts between KO HFD and WT HFD animals to identify differential Canonical Pathways due to galectin-3 ablation in addition to the response to HFD.

### Real-time PCR

Microarray results were confirmed by semi-quantitative real-time PCR (qPCR) performed on 3 biological replicate experiments. Synthesis of cDNA was obtained by reverse transcription of 1 μg of amplified RNA from the sorted endothelial cells using qScript (Quanta Biosciences, Gaithersburg, MD). Oligonucleotide primers were designed to generate amplicons 100–200 nucleotides long which spanned at least one intron. Primer sequences are listed in Additional file 2. The cDNA representing 5 ng of total RNA was amplified by PCR performed using SYBR® green fluorophore (Roche, Indianapolis, IN) in an Applied Biosystems® 7900HT fast real-time PCR system. A standard two-phase reaction (95 °C 15 sec, 60 °C 1 min) worked for all amplifications. Dissociation curves run for each reaction verified the presence of a single amplicon peak. Amplicons were also sequenced by 3730XL DNA Analyzer (Applied Biosystems), and BLAST was used to verify the alignment of the amplicon sequence with that of the target transcript (Additional file 2).

Fold changes represent the ratio of HFD to chow diet expression values determined from the cycle times where C_T_, the threshold intensity, was exceeded. The abundance of Cyclophilin A was assessed in parallel as a loading control to which the genes of interest were normalized.

### Prothrombin time

After 8 weeks of high-fat diet, blood was collected by cardiac puncture following CO_2_ asphyxiation into tubes containing sodium citrate anticoagulant at a ratio of 9:1. Plasma was separated by performing two centrifugation steps at 5000 rpm for ten minutes each. Prothrombin time was assessed using a Diagnostica Stago STArt 4 Hemostasis Analyzer according to the manufacturer’s protocol. Immediately before measurement, plasma was diluted 1/5 with Stago diluent buffer.

### GLUT4 immunofluorescence

Animals were sacrificed after 8 weeks on the diet regimen and perfused with PBS. Segments of the thoracic aorta and the entire skeletal muscle were immediately frozen in TissueTek® Optimal Cutting Temperature Compound on dry ice. Ten micrometer sections were rehydrated in PBS, permeabilized with 0.3 % Triton-X-100, and incubated overnight with rabbit anti-glucose transporter GLUT4 antibody (Abcam, Cambridge, MA, ab654) at 1:50 followed by Alexa Fluor®568 goat anti-rabbit IgG (Invitrogen) at 1:800. Stained sections were mounted in fluorescence mounting medium containing DAPI. Images were collected under controlled exposure and gain settings with an Eclipse 80i (Nikon, Tokyo, Japan) with SPOT™ software (SPOT™ Imaging Solutions, Sterling Heights, MI). Six aortic and 2 skeletal muscle sections from each of 3 WT and 4 KO mice per diet group were stained for GLUT4. The mean fluorescence intensity of GLUT4 staining in the skeletal muscle was determined using ImageJ to quantify the fluorescence intensity of 20 myofibers from each mouse.

### Statistics

Statistical analyses of the metabolic parameters and GLUT4 quantification were performed using 2-way ANOVA followed by Bonferroni post-hoc tests. For repetitive measurements made over time (GTT, glucose, insulin), a 2-way ANOVA followed by Bonferroni post-hoc tests was performed at each time-point. The area under the curve (AUC) of the glucose tolerance test was determined using the statistical software in Graphpad. P-values for pAKT densitometry and prothrombin time were calculated using a two-tailed, unpaired *t*-test. Statistically significant transcriptional dysregulation after HFD was determined by applying the one sample *t*-test function within Acuity® to the [HFD/chow] fold-change results to calculate a p-value for each transcript. Transcripts differentially dysregulated by HFD in the WT and KO strains were determined by performing a two-tailed, unpaired *t*-test between the two groups.

## Results

### Metabolic characterization of the galectin-3 (−/−) type II diabetic mouse model

**Fig. 1:**
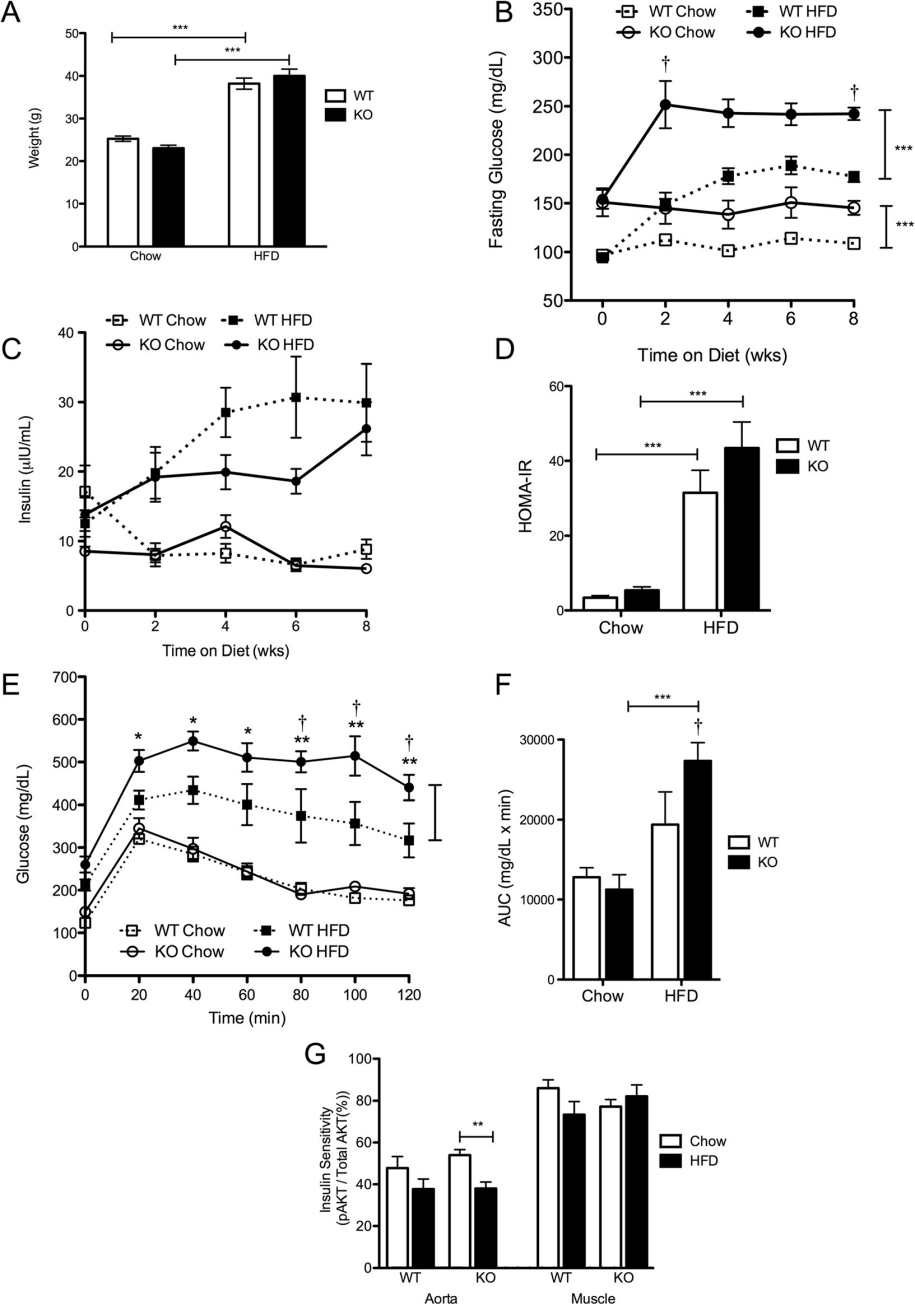
Endocrine Responses of KO vs. WT mice fed a high-fat diet. (**a**) Weight after 8 weeks of diet (HFD *n* = 19-23; Chow *n* = 16-17). Increase in fasting glucose (**b**) and insulin (**c**) levels over the time course of the diet regimen (HFD *n* = 7-26; Chow *n* = 4-22). (**d**) HOMA-IR calculated from fasting glucose and insulin levels after 8 weeks of diet *(n =* 9-10). (**e**) Glucose tolerance test performed by measuring glucose levels every 20 min after i.p. injection of glucose (1 mg/kg, *n* = 8-9) and (**f**) corresponding area under the glucose tolerance curve. (**g**) Sensitivity of aortic and skeletal muscle tissues to insulin challenge (60 U/kg) after 8 weeks of high-fat or chow diet measured by western blotting for phosphorylated AKT. Densitometry was performed using Image J, and pAKT levels are expressed as a percentage of total AKT levels (*n* = 3-8). Data is shown as mean ± SEM. *P < 0.05;**P < 0.01; ***P < 0.001; ^†^ *P* < 0.05 for the interaction of diet and genotype determined by 2-way ANOVA

As expected, both KO and WT mice on HFD have significant weight gain compared to chow-fed controls, and this weight gain is similar between the strains (Fig. 1a). Before the onset of the diet regimen, it was evident that *Lgals3 (−/−)* mice have higher fasting glucose levels than WT (153 ± 51 mg/dL vs. 95 ± 15 mg/dL, mean ± SD, *n* = 21–32, *P* < 0.0001). During the first 2 weeks of high-fat feeding, KO mice displayed a sharp increase in fasting glucose levels, which remained high for the duration of the study; whereas fasting glucose levels of WT mice on HFD increased at a steady rate, reaching statistical significance at 4 weeks, and then leveled off (Fig. 1b). By the completion of the 8 week diet regimen, fasting glucose of the KO was substantially higher than that of the WT on HFD (Fig. 1b).

Fasting insulin levels were consistently lower in the KO over the time course of the study until the 8 week time-point (Fig. 1c). Due to higher glucose levels and similar or lower insulin levels, homeostatic model assessment of insulin resistance (HOMA-IR) is higher in KO vs. WT on chow and HFD, but not significantly (Fig. 1d). After 5 weeks of HFD, both WT and KO mice have significantly impaired glucose tolerance compared to chow-fed controls as shown by the GTT, but this impairment is more severe in the KO (Fig. 1e, f).

Furthermore, exposure to HFD for 8 weeks does not result in a reduced response to insulin as reflected by similar levels of skeletal muscle AKT phosphorylation following an *in vivo* insulin challenge in both KO and WT mice on HFD (Fig. 1g, Additional file 3). However, a greater reduction in phosphorylated AKT levels was observed in aortic tissue lysates from *Lgals3 (−/−)* mice after HFD, indicating the onset of vascular insulin resistance (Fig. 1g). After 8 weeks of high-fat feeding, the level of circulating AGEs in the blood of HFD mice remain similar to that of chow-fed controls. Furthermore, there is no difference in AGE levels between the two strains [KO HFD: 1.8 ± 0.7 ng/mg protein; KO Chow: 1.7 ± 0.3 ng/mg protein; WT HFD: 1.7 ± 0.3 ng/mg protein; WT Chow: 1.7 ± 0.3 ng/mg protein (mean ± SD, *n* = 9 for HFD and *n* = 7 for chow)].

### Endothelial cell isolation

**Fig. 2:**
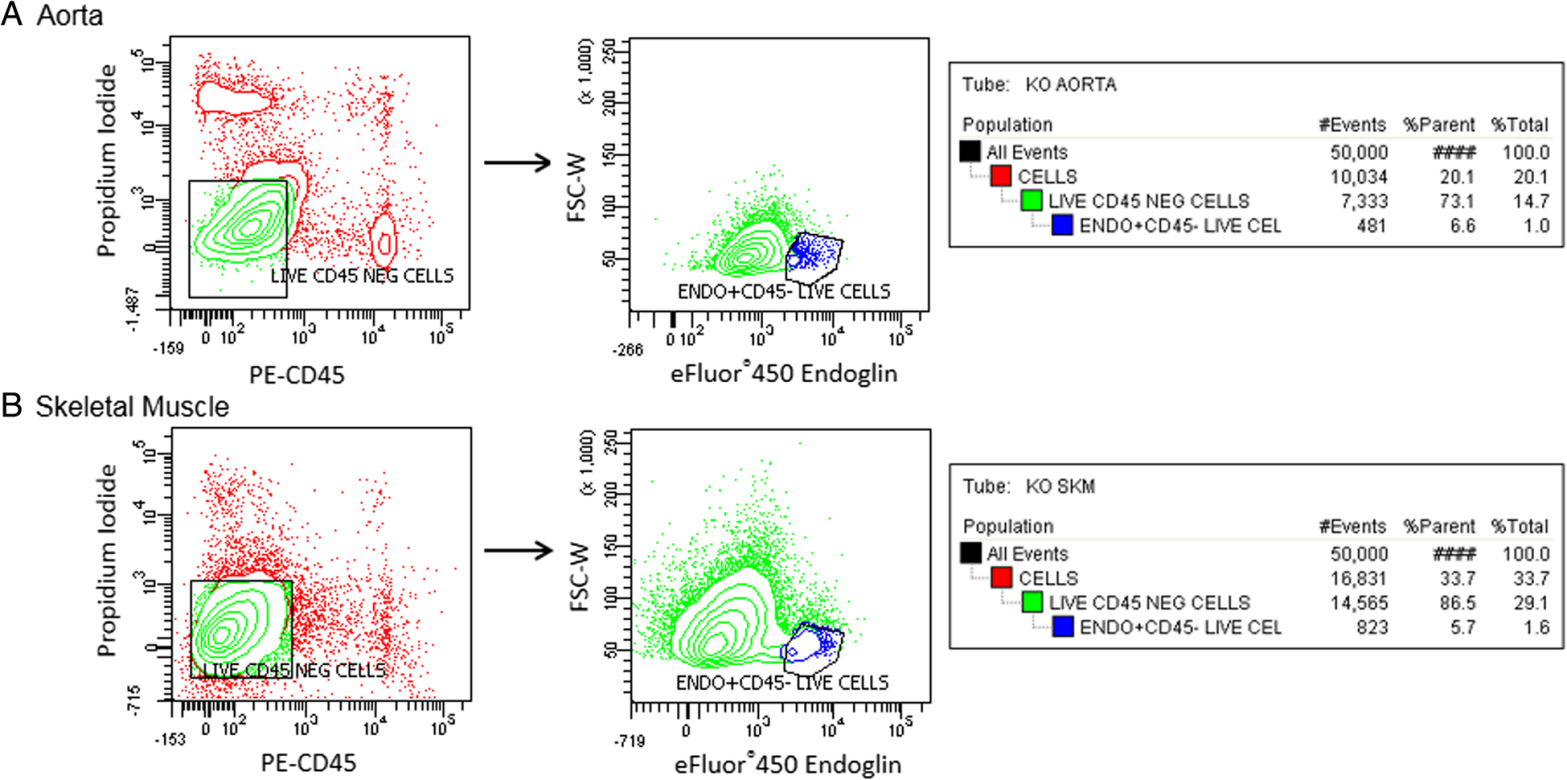
FACS isolation of Galectin-3 (−/−) endothelium. a Collagenolytic digests of aortae (**a**) and leg muscles (**b**) of Galectin-3 (−/−) and C57BL/6 mice were labeled with eFluor450-Endoglin, PE-CD45, and live/dead stain. Live, Endoglin^+^/CD45^−^ cells were sorted directly into TRIzol reagent for subsequent gene expression analysis

Endothelial cells were sorted by FACS based on positive expression of the endothelial cell-surface glycoprotein, endoglin (CD105). Endoglin is a component of the TGF-β receptor complex and is important in angiogenesis [26]. While predominantly expressed in endothelial cells, endoglin may also be expressed on activated monocytes and tissue macrophages [27]. Therefore, we also stained for the common leukocyte antigen, CD45, and excluded all CD45^+^ cells from our sorting gate. FACS sorting yielded approximately 10,000 CD105^+^/CD45^−^, live cells from each pooled sample. Figure 2 shows representative FACS profiles of skeletal muscle and aortic tissue digests, with the sorted endothelial cell population representing 5.7 % and 6.6 % of the live, CD45^−^ cell populations.

Using this same technique and applying the same sorting gate on GFP^+^ endothelial cells from Tie2-GFP mice showed that approximately 75 % of GFP^+^ cells also display a high signal for CD105. The specificity of the endoglin antibody was also tested on C2C12 muscle cells, which showed no endoglin positive cells. This technique was much more specific than sorting based on CD31 expression and stained a greater number of GFP^+^ endothelial cells than TIE2 or CD146 antibodies tested. Additional files 4 and 5 show FACS profiles of the sorted endothelial population as well as negative controls and testing of endoglin specificity.

### Transcriptional results and pathway analysis

Tables 1 and 2 display fold-change of selected transcripts with |log_2_ fold-change [HFD/chow]| >1 in all 3 distinct aortic or skeletal muscle endothelial hybridization experiments for both strains. These transcripts were selected based on level of dysregulation, or for their roles in diabetic or cardiovascular pathophysiology, or known connection to galectin-3. Complete microarray data may be found in the Gene Expression Omnibus under record number GSE57329.

**Table 1:** Aortic endothelial responses of KO and WT mice after 8 weeks of HFD vs. chow

Genbank accession	Gene name	WT H/C	KO H/C
NM_007606	carbonic anhydrase 3	−3.66	−3.81
NM_007617	caveolin 3	1.49	2.91
NM_007728	coagulation factor C homolog	−1.42	−2.07
NM_007736	collagen, type IV, alpha 5	0.68	2.17
NM_007925	Elastin	1.06	1.79
NM_007940	epoxide hydrolase 2, cytoplasmic	−0.84	−1.42
NM_008764	tumor necrosis factor receptor superfamily, member 11b (osteoprotegerin)	1.32	4.04
NM_008966	prostaglandin F receptor	0.98	2.01
NM_009204	solute carrier 2a4 (Glut4)	−1.06	−2.19
NM_009325	thromboxane A2 receptor	1.19	1.43
NM_009605	adiponectin, C1Q and collagen domain containing	−5.47	−4.11
NM_009928	collagen, type XV, alpha 1	1.95	2.96
NM_010171	coagulation factor III	0.83	1.58
NM_010570	insulin receptor substrate 1	0.51	1.34
NM_010766	macrophage receptor with collagenous structure	1.07	4.14
NM_011101	protein kinase C, alpha	0.75	2.39
NM_011580	thrombospondin 1	−1.71	−0.20
NM_013459	complement factor D (adipsin)	−4.26	−4.02
NM_018762	glycoprotein 9 (platelet)	−0.50	−2.77
NM_020509	resistin like alpha	−4.44	−2.45
NM_021282	cytochrome P450, family 2, subfamily e, polypeptide 1	−4.50	−4.79
NM_022984	Resistin	−4.27	−4.49
NM_026280	matrix-remodelling associated 7	1.27	1.94
NM_028784	coagulation factor XIII, A1 subunit	−2.32	−0.97
NM_053185	collagen, type IV, alpha 6	0.76	1.75
NM_153526	insulin induced gene 1	−0.30	−1.34
NM_178793	collagen and calcium binding EGF domains 1	0.07	1.48

Transcripts shown are dysregulated > 1log_2_ [fold change] in either KO or WT compared to their respective chow-fed controls

**Table 2:** Skeletal muscle endothelial responses of KO and WT mice after 8 weeks of HFD vs. chow

Genbank accession	Gene name	WT H/C	KO H/C
NM_008491	lipocalin 2	−1.23	−2.06
NM_011784	apelin receptor	1.56	1.52
NM_009325	thromboxane A2 receptor	1.78	1.74
NM_019985	C-type lectin domain family 1, member b	0.64	−1.03
BC007177	cyclin L1	−0.06	−1.15
NM_010104	endothelin 1	−0.48	−1.15
AK042211	endothelin receptor type A	−0.85	−1.44
NM_009605	adiponectin, C1Q and collagen domain containing	0.26	−1.16
NM_021896	guanylate cyclase 1, soluble, alpha 3	−1.31	−1.16
NM_009694	apolipoprotein B mRNA editing enzyme, catalytic polypeptide	0.58	−1.18
NM_026672	glutathione S-transferase, mu 7	−0.75	−1.32
NM_007413	adenosine A2b receptor	−0.49	−1.35
NM_177687	cAMP responsive element binding protein-like 2	−1.23	−1.67
NM_008161	glutathione peroxidase 3	−1.84	−1.68
NM_053247	lymphatic vessel endothelial hyaluronan receptor 1	0.90	−1.20
NM_009805	CASP8 and FADD-like apoptosis regulator	0.39	−1.02
NM_013459	complement factor D (adipsin)	−1.77	−3.65
NM_009928	collagen, type XV, alpha 1	2.59	2.27
NM_011607	tenascin C	2.00	1.11
NM_021281	cathepsin S	3.30	1.45
X70100	fatty acid binding protein 5, epidermal	1.47	0.94
L38613	glucagon receptor	1.39	0.17
J05020	Fc receptor, IgE, high affinity I	1.37	1.15
NM_011311	S100 calcium binding protein A4	1.30	2.01
NM_008332	interferon-induced protein with tetratricopeptide repeats 2	1.05	0.50
NM_008329	interferon activated gene 204	1.18	0.65
NM_008620	guanylate binding protein 4	1.05	0.52
AK087208	endothelial PAS domain protein 1	−1.06	−0.78
NM_178020	hyaluronoglucosaminidase 3	−1.11	−0.20
AK032692	immediate early response 2	−1.18	−0.36
AK163452	aldehyde dehydrogenase 2, mitochondrial	−1.33	−0.55
NM_133808	high density lipoprotein (HDL) binding protein	−0.57	0.05
NM_027286	Angiotensin I converting enzyme	−1.36	−0.58

Transcripts shown are dysregulated >1log_2_ [fold change] in either KO or WT compared to their respective chow-fed controls

Data analysis using IPA® revealed “Cardiovascular System Development and Function” as a top over-represented biological function with dysregulated transcripts presented in Additional file 6. Analysis of differentially regulated aortic endothelial transcripts between KO HFD and WT HFD mice revealed the “Coagulation Cascade” to be a highly up-regulated pathway in the KO HFD endothelium. Log_2_ fold change [KO HFD/WT HFD] of transcripts belonging to this canonical pathway are shown in Additional file 7.

**Fig. 3:**
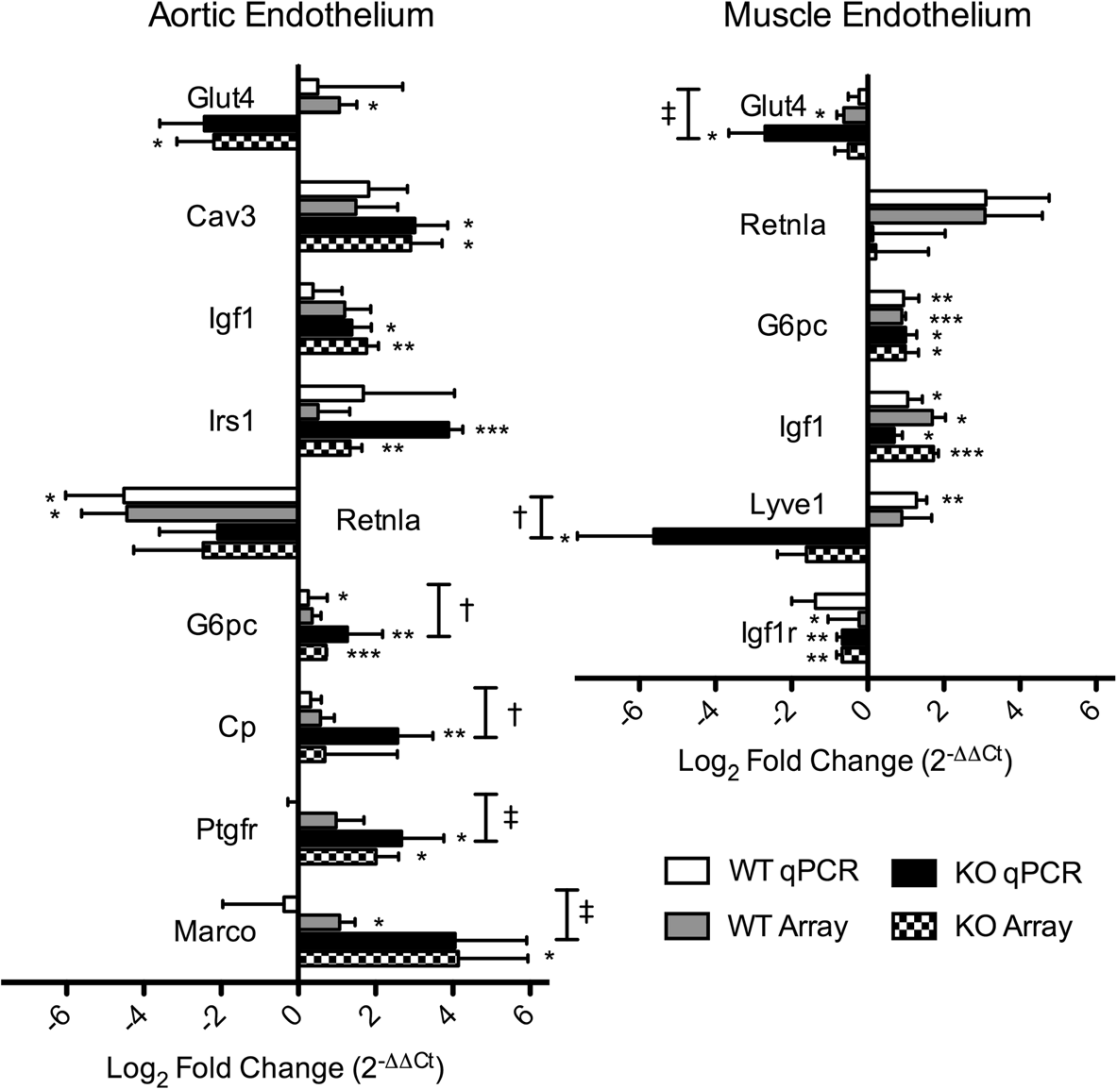
Log_2_ fold change of endothelial transcripts dysregulated by HFD in KO and WT mice**.** Log_2_ fold change of endothelial transcripts dysregulated by high-fat diet vs. chow diet in the aortic endothelium (**a**) and skeletal muscle endothelium (**b**) of Galectin-3 (−/−) and WT mice after 8 weeks of feeding were determined by qPCR and microarray analyses. Data is presented as mean + SEM. *P ≤ 0.1, **P < 0.05, and ***P < 0.01 by one sample *t*-test. ^‡^ *P* ≤ 0.1 and ^†^ *P* < 0.05 vs. WT

The expression levels of selected dysregulated transcripts were evaluated by qPCR. These genes include caveolin-3 (*Cav3*), insulin receptor substrate-1 *(Irs-1*), glucose-6-phosphatase (*G6pc*), the glucose transporter *Glut4* (*Slc2a4*), resistin-like alpha (*Retnla*), prostaglandin F receptor (*Ptgfr*), ceruloplasmin (*Cp)*, macrophage receptor with collagenous structure (*Marco*), lymphatic vessel endothelial hyaluronan receptor 1 (*Lyve1*), and insulin-like growth factor 1 (*Igf1)* and its receptor, *Igf1r*. The real-time PCR expression levels are presented in Fig. 3 as log_2_ fold change [HFD/control] for KO and WT strains. The corresponding fold-changes derived from the microarray data are also shown for comparison.

### Physiological assessment of coagulation pathway activity

**Fig. 4:**
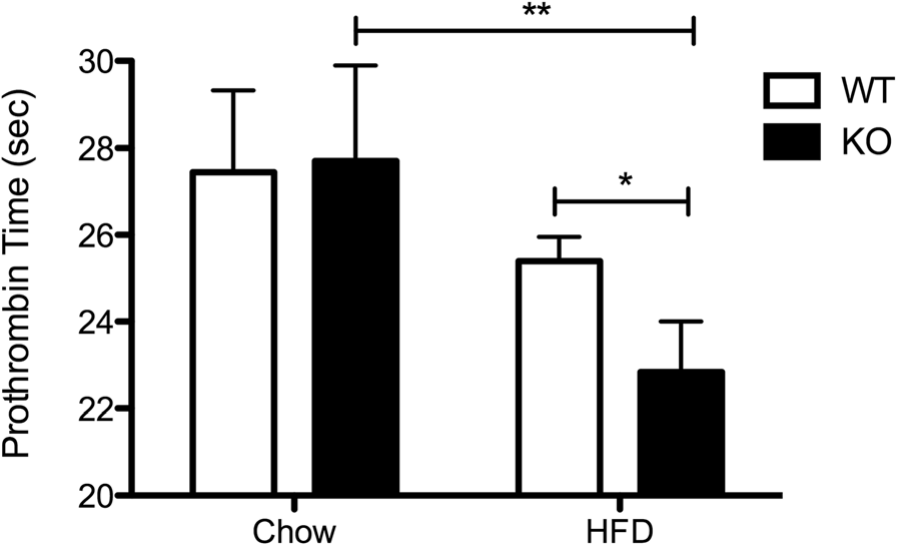
Activation of the extrinsic coagulation pathway in diabetic KO mice**.** Prothrombin time of citrate-anticoagulated plasma from WT and KO mice fed a high-fat or chow diet for 8 weeks was assessed by measuring the time to coagulation following the addition of a calcium thromboplastin reagent using a Diagnostica Stago Start 4 Hemostasis Analyzer. *P <0.05; **P <0.01. *N* = 3 WT animals and *n* = 4-5 KO animals

Due to the up-regulation of transcripts involved in the coagulation pathway in the aortic endothelium from KO diabetic mice compared to WT diabetic mice, coagulation activity was assessed in the plasma after 8 weeks of high-fat or chow diet. While no difference in prothrombin time (PT) was observed between chow-fed KO and WT animals, high-fat-fed KO animals displayed reduced PT compared to chow-fed controls and high-fat-fed WT mice (Fig. 4).

### GLUT4 expression in skeletal muscle and aortic endothelium

**Fig. 5:**
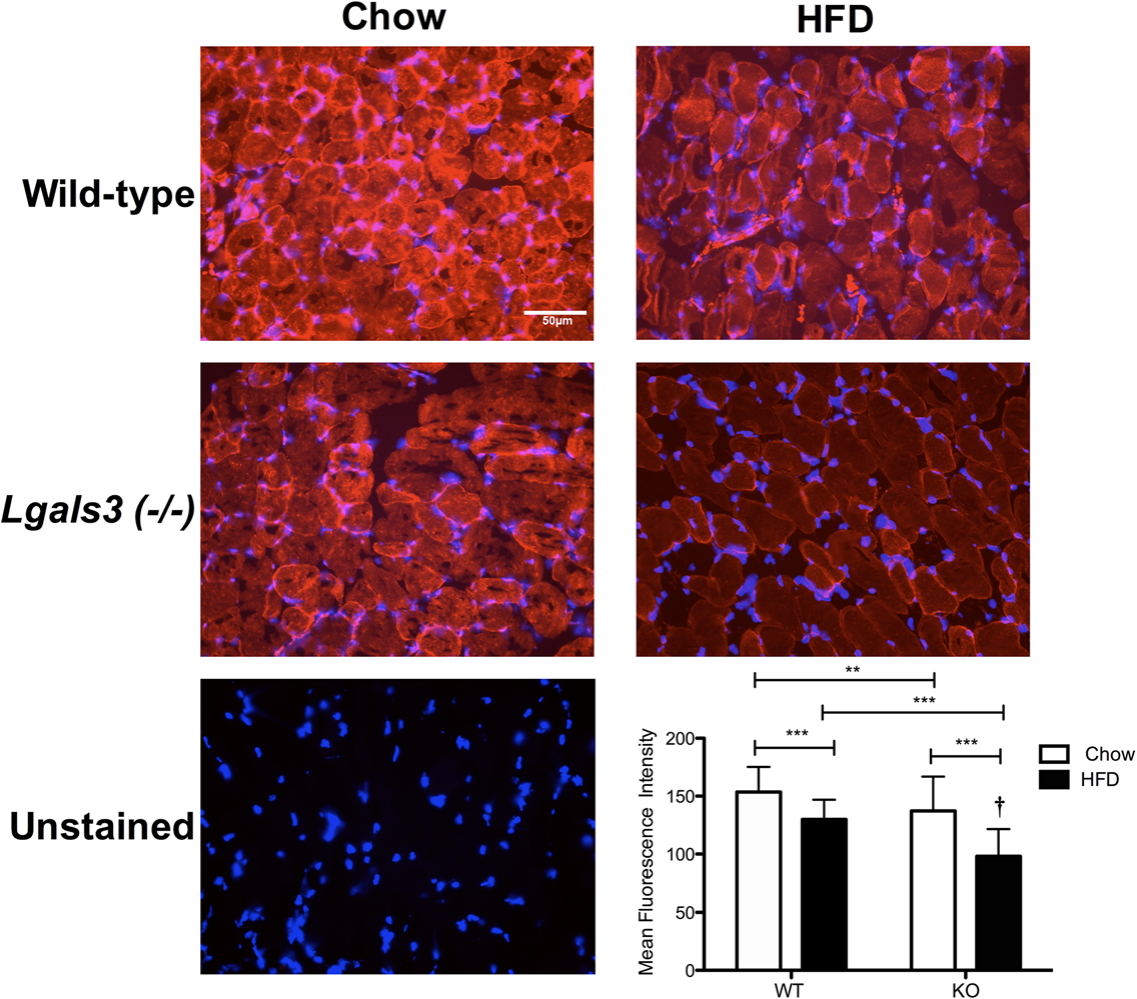
Immunofluorescence staining for GLUT4 protein in the skeletal muscle of WT and KO mice. Ten micrometer, fresh-frozen sections from KO and WT mice fed a high-fat or chow diet for 8 weeks were incubated overnight with rabbit anti-mouse GLUT4 antibody followed by incubation with an Alexa Fluor®568-conjugated goat anti-rabbit IgG secondary antibody followed by mounting in fluorescence mounting medium containing DAPI. Representative images from 3 WT and 4 KO mice per group are shown at 40X magnification. Primary antibody was omitted from the unstained control. Mean fluorescence intensity of 20 myofibers from 2 sections per animal was quantified using Image J. **P < 0.01; ***P <0.001 by 2-way ANOVA followed by Bonferroni post-hoc tests. ^†^ *P* < 0.05 for the interaction of diet and genotype by 2-way ANOVA

**Fig. 6:**
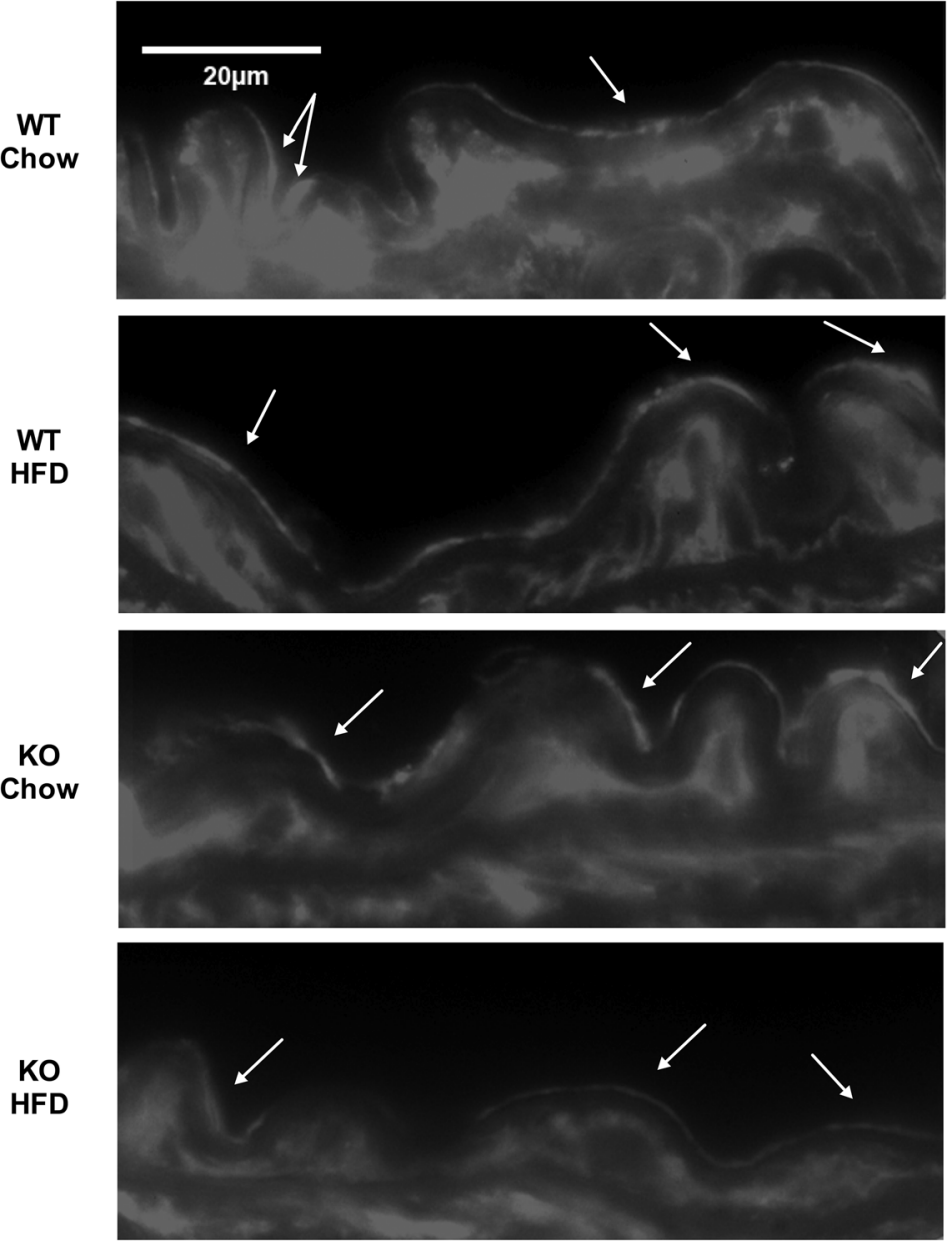
Immunofluorescence staining for GLUT4 protein in aortic cross-sections from WT and KO mice. Ten micrometer, fresh-frozen sections from KO and WT mice fed a high-fat or chow diet for 8 weeks were incubated overnight with rabbit anti-mouse GLUT4 antibody followed by incubation with an Alexa Fluor®568-conjugated goat anti-rabbit IgG. Representative images from 3 WT and 4 KO mice per group are shown at 100X magnification in grayscale. Arrows indicate areas of GLUT4 staining in the endothelial cell cytoplasm. No staining was observed in unstained controls where primary antibody was omitted (not shown)

Immunofluorescence staining for GLUT4 in skeletal muscle sections from chow- and HFD-fed WT and KO mice, revealed reduced protein expression in both strains after 8 weeks of high-fat feeding compared to chow-fed controls (Fig. 5). In chow-fed animals, intense GLUT4 fluorescence was localized to the plasma membrane with staining in the cytosol as well (Fig. 5). KO mice on either diet displayed less GLUT4 fluorescence compared to the WT strain. Diabetic KO mice had greatly reduced levels of GLUT4, especially at the membrane, compared to all other groups (Fig. 5). In the aortic endothelium, similar levels of GLUT4 were observed in chow and HFD WT animals (Fig. 6). However, KO mice fed a high-fat diet had less endothelial GLUT4 compared to chow-fed KO mice and WT mice on either diet (Fig. 6).

## Discussion

Galectin-3 (−/−) mice display greater metabolic derangement in response to HFD compared to WT mice, including both extreme hyperglycemia and impaired response to glucose challenge (Fig. 1). Our study indicates that the elevated glucose levels observed in the galectin-3 knockout may be explained by impaired glucose utilization due to reduced levels of GLUT4. Our transcriptional data revealed exacerbated down-regulation of *Glut4* mRNA in the KO, by more than 2-fold in the aortic endothelium and 4-fold in the muscle endothelium compared to WT (Fig. 3). Immunofluorescence confirmed the reduced abundance of GLUT4 protein in both tissues of the KO after high-fat feeding (Figs. 5, 6).

Interestingly, chow-fed KO mice display isolated hyperglycemia, which may be useful to elucidate pathways specifically related to increased glucose. Chow-fed KO mice also show reduced abundance of GLUT4 protein relative to WT chow animals. Since GLUT4 is the primary transporter responsible for glucose uptake into insulin-sensitive tissues such as muscle and fat, reduced levels of GLUT4 likely contributes to the hyperglycemia and metabolic dysfunction observed in the KO.

The down-regulation of *Glut4* expression has been reported in several models of diabetes and cardiovascular disease. It has long been known that high-fat feeding reduces GLUT4 expression in insulin-sensitive tissues, such as skeletal muscle and fat [28]. In a rat model of type II diabetes induced by high-fat feeding and streptozotocin injection, cardiac GLUT4 protein was reduced, likely contributing to the decrease in glucose metabolism in the diabetic heart [29]. Isoproterenol treatment has also been shown to decrease *Glut4* expression, while treatment with the dipeptidyl peptidase 4 inhibitor, vildagliptin, improved *Glut4* mRNA expression [30]. In the aortae of angiotensin II hypertensive mice, GLUT4 expression was reduced by 46 % [31]. Gaudreault et al. observed more than 50 % reduction in GLUT4 protein in *en face* preparations of coronary artery endothelial cells from STZ-treated rats exposed to hyperglycemia for 8 weeks [32]. They also showed altered localization of GLUT4 from the abluminal to the luminal side of the endothelial cell derived from diabetic rats [32].

Studies of the *Glut4* promoter have identified “high-fat responsive elements” and PPARγ regulation of transcription in adipocytes [33, 34]. In muscle, *Glut4* expression is regulated by several factors including myocyte enhancer factor 2 (MEF2), GLUT4 enhancer factor (GEF), MyoD myogenic proteins, thyroid hormone receptors, and Kruppel-like factor 15 (KLF15) [35, 36]. These studies demonstrate that GLUT4 expression is subject to metabolic and tissue-specific regulation. To our knowledge, we are the first to report a link between galectin-3 and the expression of GLUT4. Further studies are necessary to understand the molecular basis of the expression of *Glut4* in endothelial cells and the role of galectin-3 in the regulation of *Glut4* expression.

Our studies show that galectin-3 ablation results in the down-regulation of *Glut4* transcription as well as decreased abundance of GLUT4 protein. GLUT4 immunofluorescence in skeletal muscle sections appears to indicate a reduced localization of GLUT4 to the cell membrane in galectin-3 (−/−) mice compared to WT. However, further studies are necessary to determine if galectin-3 directly regulates the cell surface expression of GLUT4 and thereby affects glucose flux across the cell.

It is possible that the elevated glucose levels in the KO are due to hepatic insulin resistance, which may lead to increased gluconeogenesis and glycogenolysis. However, a recent study did not find any up-regulation of proteins involved in gluconeogenesis in the liver of the KO vs. WT mice [37]. It is also possible that the dysregulation of other glucose transporters contributes to the observed hyperglycemia. Our transcriptional data also revealed the down-regulation of the insulin-independent glucose transporter, GLUT1, in the aortic endothelium of the KO, but to a lesser degree than GLUT4 and not significantly different from its down-regulation in WT animals. However, it is possible that its expression is reduced in other tissues, contributing to decreased glucose uptake. We did not find altered expression of GLUT1 in the muscle endothelium, but its expression in whole muscle tissue of the KO has not been investigated. Similarly, a recent study on the visceral adipose tissue of the KO did not observe differential expression of GLUT1 [37].

While WT mice seem to maintain lower glucose levels by compensating with increased insulin output, insulin secretion in the KO is not proportionally increased (Fig. 1c). Interestingly, pancreatic insulitis has been observed in KO mice on chow and HFD, and galectin-3 over-expression has been shown to protect B-cells against toxicity [38, 37]. It is possible that beta cell destruction or dysfunction results in reduced insulin production and/or secretion, which also affects the ability of the KO to regulate blood glucose.

By 5 weeks of HFD, both WT and KO animals display impaired glucose tolerance as demonstrated by GTT (Fig. 1e). While first phase insulin release is delayed in both strains, there is a prolonged second phase insulin response in diabetic KO animals vs. WT animals, suggesting that KO mice may be experiencing greater peripheral insulin resistance. After 8 weeks of HFD, peripheral insulin resistance in the skeletal muscle is not seen in either KO or WT mice on HFD, as demonstrated by similar levels of AKT phosphorylation compared to chow-fed controls (Fig. 1g). However, vascular insulin resistance is observed in the HFD-fed KO after this diet duration (Fig. 1g), and it is possible that muscle insulin resistance may also have earlier onset in the KO.

Interestingly, circulating levels of advanced glycation endproducts (AGEs) are similar between both strains and are not elevated by 8 weeks of high-fat diet. Therefore, the differential responses we observe between the KO and WT are likely to be independent of galectin-3’s role in the uptake of AGEs. Previous studies have attributed the protective effects of galectin-3 in diabetes and atherosclerosis to an indirect role in the removal of AGEs from circulation, thereby preventing subsequent tissue damage and activation of the pro-inflammatory RAGE-pathway [14, 39]. Our data suggests a direct involvement of galectin-3 in the regulation of glucose metabolism and vascular pathology.

Our assessment of the endothelial response to diabetes in KO and WT mice has shown a greater transcriptional response in the aortic endothelium of KO mice, with twice as many dysregulated transcripts compared to WT. Transcripts with recognized roles in metabolic signaling, ECM synthesis, vasoconstriction, coagulation, and inflammation are more dysregulated in the aortic endothelium of the KO compared to WT (Fig. 3, Table 1). For example, the glucose transporter, *Glut4*, is down-regulated by HFD in both strains, but its reduction in the KO is twice as great as WT (Fig. 3). Similarly, expression of *Irs-1*, insulin-induced gene 1, *Igf1*, and glucose-6 phosphatase are dysregulated to a greater degree in the KO vs. WT on HFD (Fig. 3). The exacerbated response of the KO aortic endothelium to HFD suggests a greater derangement of metabolic signaling in these cells and implicates LGALS3 in the preservation of such pathways.

In the capillary endothelium of the skeletal muscle, there was less dysregulation by HFD in both strains compared to the aortic transcriptional response, suggesting a greater role for galectin-3 in the macrovascular complications of diabetes compared to microvascular disease. However, transcripts with roles in glucose homeostasis, atherosclerosis, and matrix remodeling are differentially regulated by HFD in the KO microvasculature vs. WT (Table 2). Transcripts which serve a protective role in glucose homeostasis, such as *Glut4* and the adenosine A2 receptor, are preferentially down-regulated. Adenosine A2 receptor knockout mice exhibit impaired glucose tolerance and insulin signaling [40].

Several ECM components and matrix remodeling proteins are also dysregulated in the diabetic KO endothelium (Fig. 3, Tables 1 and 2). In the muscle endothelium, a receptor for hyaluronic acid (HA), *Lyve1*, is downregulated 6-log_2_ fold (Fig. 3). *Lyve1* is expressed in lymphatic endothelial cells and is involved in the degradation of HA, a matrix component that facilitates cell migration during wound healing and inflammation [41]. The downregulation of *Lyve1* suggests decreased HA turnover which could impair wound healing in the diabetic KO.

Transcripts implicated in atherosclerosis are also dysregulated in the KO endothelium. HDL binding protein, which is normally expressed by endothelial cells, is down-regulated by HFD in the WT but not the KO. This protein was up-regulated in atherosclerotic lesions in human coronary arteries [42]. Similarly, transcriptional up-regulation of the scavenger receptor, *Marco*, in the aortic endothelium of the diabetic KO by more than 4-log_2_ fold over chow-fed controls was observed by array and qPCR (Fig. 3). This receptor serves a role in the binding and uptake of modified LDL and has been shown to be constitutively expressed in endothelial cells of the lymph nodes [43].

Pathway analysis of the HFD-fed KO and HFD-fed WT transcriptional profiles revealed the up-regulation of transcripts involved in the coagulation cascade in the KO endothelium (Additional file 7). Our physiological assessment of coagulation activity confirmed that diabetic KO mice exhibit reduced prothrombin time compared to diabetic WT mice and chow-fed controls (Fig. 4). Prothrombin time specifically measures activation of the extrinsic coagulation pathway activated by tissue factor. Therefore, the diabetic KO displays increased activation of the extrinsic coagulation pathway, which has important implications for diabetic patients where increased activation of the coagulation cascade could promote thrombotic complications.

Furthermore, the transcript for von Willebrand factor (vWF), a protein important in platelet activation and aggregation, is also up-regulated in the endothelium of HFD KO mice compared to HFD WT mice (Additional file 7). Recent studies have shown that LGALS3 and LGALS1 interact directly with vWF in endothelial cells and in plasma via the N-linked glycans of vWF [44]. The inhibition of these galectins was associated with increased vWF-platelet string formation and more rapid thombus formation after injury [44]. Our data suggests that LGALS3 may protect against thrombus formation in diabetes by suppressing vWF expression in the endothelium.

## Conclusions

Our analysis of the transcriptional response of the endothelium to an *in vivo* model of type II diabetes in galectin-3 deficient vs. wild-type mice has revealed differential responses in both aortic and skeletal muscle tissues. The KO displays altered expression of transcripts with roles in the glucose-insulin signaling pathway, ECM composition, vasoregulation, redox homeostasis, inflammation, coagulation, atherosclerosis, and endothelial dysfunction. Down-regulation of GLUT4 mRNA and protein in the endothelium and muscle of galectin-3 (−/−) mice may explain the hyperglycemia experienced by these mice, and suggests a role for galectin-3 in the regulation of glucose uptake. Transcriptional up-regulation of coagulation and pro-thrombotic factors and increased blood clotting activity in the plasma from diabetic KO mice also suggests a protective role for galectin-3 in mediating coagulation and thrombosis. The transcriptional findings, as well as the altered metabolism demonstrated by KO mice compared to WT mice, suggest that galectin-3 serves a protective role against the metabolic and hormonal derangements that impinge upon the diabetic endothelium and lead to its damage and dysfunction.

## Additional files

Additional file 1:
**Confirmation of galectin-3 deletion in the knockout mouse at the DNA, RNA, and protein level.**
Additional file 2:
**Sequences of primers used for the real-time PCR experiments and the resulting amplified products.**
Additional file 3:
**Insulin sensitivity of aortic and skeletal muscle tissues after 8 weeks of high-fat or chow diet.**
Additional file 4:
**Representative FACS profiles of the sorted endothelial population derived from the skeletal muscles and aortae of Galectin-3 (−/−) and C57BL/6 mice.**
Additional file 5:
**Specificity of endoglin antibody for binding endothelial cells in a skeletal muscle digest compared to the lack of non-specific binding in a cultured muscle cell suspension.**
Additional file 6:
**Transcripts identified by Ingenuity Pathway Analysis in the Cardiovascular Function and Development category, which was the most enriched biological function in the KO data.**
Additional file 7:
**The Coagulation Cascade is highly up-regulated in the aortic endothelium of diabetic KO mice compared to diabetic WT mice.**
