# Supplementary material for: Galectin-3 deficiency exacerbates hyperglycemia and the endothelial response to diabetes
Source: Cardiovasc Diabetol. 2015 Jun 6;14:73. doi: 10.1186/s12933-015-0230-3 (PMC4499178; doi:10.1186/s12933-015-0230-3)
Supplement: Additional file 1: — Confirmation of galectin-3 deletion in the knockout mouse at the DNA, RNA, and protein level. [file 12933_2015_230_MOESM1_ESM.pdf]

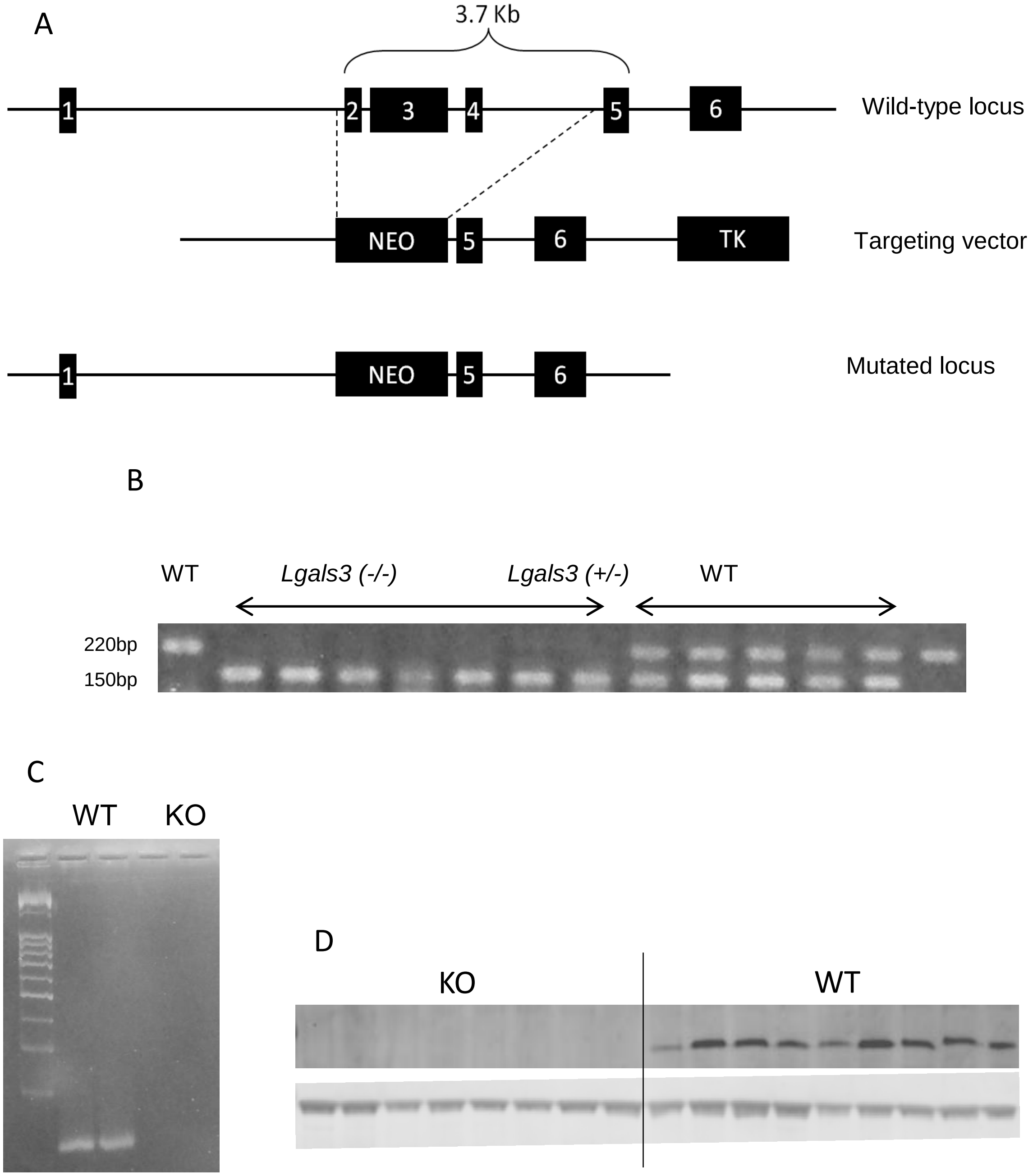

Additional File 1. Confirmation of galectin-3 deletion in the knockout mouse at the DNA, RNA, and protein level. (A) Scheme for generating the null mutation by homologous recombination (adapted from Colnot, 1998). (B) Analysis of genomic DNA revealing fragments corresponding to the wild-type and mutated alleles at 220bp and 150bp, respectively. (C) Amplified products from qPCR using *Lgals3* primers designed within the deleted region show no amplified product in KO lanes. (D) Confirmation of galectin-3 protein loss in aortic lysates resulting from gene ablation.
