# Supplementary material for: Galectin-3 deficiency exacerbates hyperglycemia and the endothelial response to diabetes
Source: Cardiovasc Diabetol. 2015 Jun 6;14:73. doi: 10.1186/s12933-015-0230-3 (PMC4499178; doi:10.1186/s12933-015-0230-3)
Supplement: Additional file 2: — Sequences of primers used for the real-time PCR experiments and the resulting amplified products. [file 12933_2015_230_MOESM2_ESM.pdf]

Additional File 2. Sequences of primers used for the real-time PCR experiments and the resulting amplified products. Following RT-PCR, amplicons were purified and sequenced by a 3730 DNA Analyzer (ABI). BLAST was used to confirm alignment of amplicon and transcript sequences.

Gene	Forward / Reverse Primers	Amplicon Sequence
Lgals3<br>NM_010705	TTT TCG CTT AAC GAT GCC TTA G<br><br>GTA GGC CCC AGG ATA AGC AG	TCGGTTTTGACAAACCCTCAGGAATCCGGGTGCATGGGG<br>GAACCAGCCTGGGGCAGGGGGCTACCCAGGGGCTGCTT<br>ATCCTGGGGCCTACA
Igf1<br>NM_010512	AGG AGA CTG GAG ATG TAC TGT GC<br><br>CTT CGT TTT CTT GTT TGT CGA TAG	CAGGTAAGCGTCAAAGCAGCCCGCTCTATCCGTGCCCAG<br>CGCCACACTGACATGCCCAAGACTCAGAAGTCCCCGTCC<br>CTATCGACAAACAAGAAAACGAAGAAA
G6pc<br>NM_175935	GGG TCT TCC TCT TAG CAC ATT TC<br><br>AGC AGT CAA CCC ATA AAA GCT AAG	CCCAGTGCTGGGCGGCTTATTGTTGGTGCTGCCCTTGGC<br>TGGCTAATGAGCCCCCGGGTACCCATGGAGCGGGAGCT<br>TAGCTTTTATGGGTTGACTGCTA
Igf1r<br>NM_010513	GGA TGG TGT CTT CAC TAC TCA TTC T<br><br>ATG ACG AAA CGA AGA ACT TGC T	GTGGGTTCTGTCTTCGGGTCGTCTCTGGGAGTCGCCACG<br>CTGGCTGAGCAGCCCTACCAGGGCTTGTCCAACGAGCAA<br>GTTCTTCGTTTCGTCATCGCAAGTTCTTCGTTTCGTCATA
Lyve1<br>NM_053247	AAA CAG AAG CAT TTG TTG CAA GT<br><br>CTT CAC ATA CCT TTT CAC GTA GCA	CGGGACAGTTTCAAGAACGAAGCAGCTGGGTTTGGAGGT<br>GTCCCCACCGCCCTGCTGGTGCTGGCTCTCCTCTTCTTT<br>GGTGCTGCCGCTGTGCTGGCTGTTTGCTACGTGAAAAGG<br>TATGTGAAGA
Retnla<br>NM_020509	AAG ACT ACA ACT TGT TCC CTT CTC A<br><br>TTC GTT ACA GTG GAG GGA TAG TTA G	TTTCCTTTCCCTGCTCAGCTGATGGTCCAGTGATACTGAT<br>GAGACCATAGAGATTATCGTGGAGAATAAGGTCAAGGAA<br>CTTCTTGCCAATCCAGCTAACTATCCCTCCACTGTAACGA<br>AAA
Cp<br>NM_007752	GTT CTA CTT GTT TCC CAC AGT GTT T<br><br>GGA GTG CAT CTT ATT AGA CTC CTG A	AGGAAGATGAGAGTTACTCTTAGATGATAATATCAGGATG<br>TTCACAACTGCACCTGATCAAGTGGATAAGGAAGATGAA<br>GACTTTCAGGAGTCTAATAAGATGCACTCCA
Cav3<br>NM_007617	GGC TTT GCG TTC ACA TGT ACT<br><br>GCA GTT AAA ACC CTT TAT TGC AG	GGGGAAGCCATTTTCAAAAGGTGTGATCGTCTCCTCATG<br>CTAGGGGGAGCAAGCAGTGACTGCCGGCATTGAGGAAG<br>GTGCTGCAATAAAGGGTTTTAACTGCAACCCA
Glut4<br>NM_009204	GGC ATC AAT GCT GTT TTC TAC TAT T<br><br>TCT ACT AAG AGC ACC GAG ACC AAC	ATCATTTTGAGTCGGCTGGGGTGGGAAGCCAGCCTACGC<br>CACCATAGGAGCTGGTGTGGTCAATACGGTCTTCACGTT<br>GGTCTCGGTGCTCTTAGTAGAAA
Irs1<br>NM_010570	ACA GCA GAA TGA AGA CCT AAA TGA C<br><br>GTA CCA TCT ACT GAA GAG GAA GAC G	CCTTCCCTTTTTTAACTCATGGGTACCCAGACTCGAACTA<br>TTTCACAATTCACAACCAGGACCTCACGTCTTCCTCTTCA<br>GTAGATGGTACA
Marco<br>NM_010766	GGG ATG AAA GGG TCT TCT GG<br><br>GTC CCC CAC TCA TTG TTA TAG TAA A	TGAATCTTGTAGAAGGGTCAAAAGGCGATCTTTCCACGC<br>GTCCGGATCATGGGTGGCACCAACAGAGGCCGAGCTGA<br>AGTTTACTATAACAATGAGTGGGGGACCACAAGACCTTTT<br>CTCCCAA
Ptgfr<br>NM_008966	GTT CAG AAG CCA GCA GCA TAG<br><br>ACT GGG GAA TTA TTT CCA TTT ATT G	CTACCAGCTGGCAATGATCATTCAGCTCCTGGCCATAATG<br>TGCGTCTCCTGCGTCTGCTGGAGTCCCTTTCTGGTAACA<br>ATGGCCAACATTGCAATAAATGGAAATAATTCCCCAGT
Cyclophilin A<br>NM_008907	CAAAGTTCCAAAGACAGCAGAAAAC<br><br>GGCACATGAATCCTGGAATAATTC	GGGGGGCCCTGGAGAGAAAGGTTTGGCTATAAGGGTTC<br>CTCCTTTCACAGAATTATTCCAGGATTCATGTGCCA
