# Supplementary material for: Galectin-3 deficiency exacerbates hyperglycemia and the endothelial response to diabetes
Source: Cardiovasc Diabetol. 2015 Jun 6;14:73. doi: 10.1186/s12933-015-0230-3 (PMC4499178; doi:10.1186/s12933-015-0230-3)
Supplement: Additional file 3: — Insulin sensitivity of aortic and skeletal muscle tissues after 8 weeks of high-fat or chow diet. [file 12933_2015_230_MOESM3_ESM.pdf]

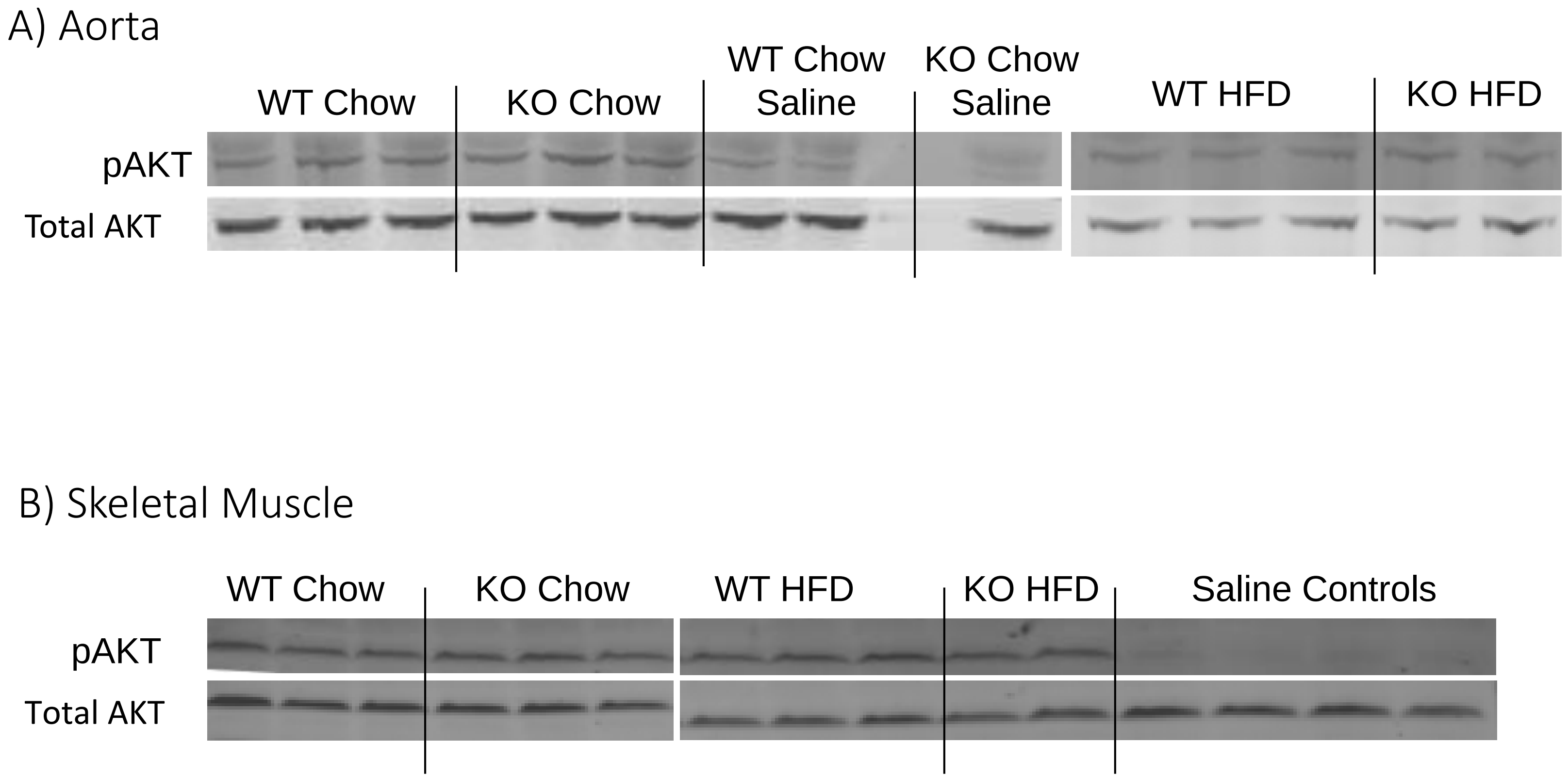

Additional File 3. Insulin sensitivity of aortic (A) and skeletal muscle (B) tissues after 8 weeks of high-fat or chow diet. Tissue lysates from animals stimulated with 60U/kg insulin or saline control were probed for phosphorylated AKT. Blots were stripped and probed for total AKT to which the quantity of pAKT was normalized. Representative blots are shown above for each tissue and the densitometry performed using ImageJ is shown in Figure 1G (*n* = 2-8 per group).
