# Supplementary material for: Galectin-3 deficiency exacerbates hyperglycemia and the endothelial response to diabetes
Source: Cardiovasc Diabetol. 2015 Jun 6;14:73. doi: 10.1186/s12933-015-0230-3 (PMC4499178; doi:10.1186/s12933-015-0230-3)
Supplement: Additional file 4: — Representative FACS profiles of the sorted endothelial population derived from the skeletal muscles and aortae of Galectin-3 (−/−) and C57BL/6 mice. [file 12933_2015_230_MOESM4_ESM.pdf]

A) Skeletal muscle

PI only skeletal muscle sample

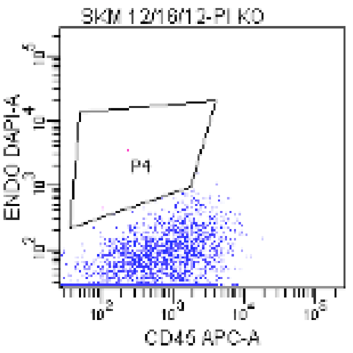

Tube: PI KO

Population	#Events	%Parent	%Total
All Events	20,164	####	100.0
SINGLETS	4,316	21.4	21.4
P1	3,685	85.4	18.3
P3	2,936	79.7	14.6
P4	2	0.1	0.0
P5	1	50.0	0.0
CELLS	3,924	90.9	19.5
CD45-PI-	2,940	74.9	14.6
ENDO+	1	0.0	0.0

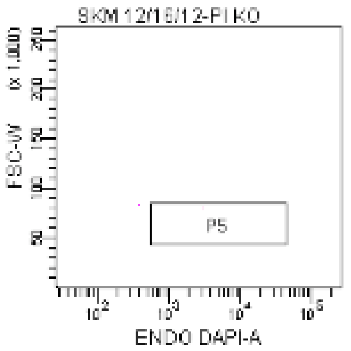

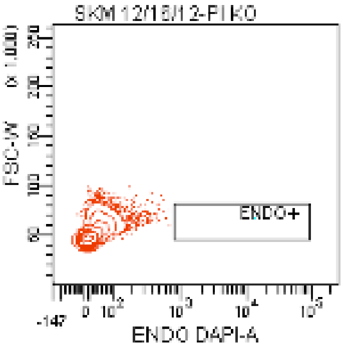

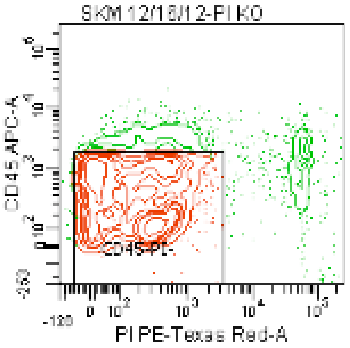

Skeletal muscle sample stained for Endoglin, CD45, & PI

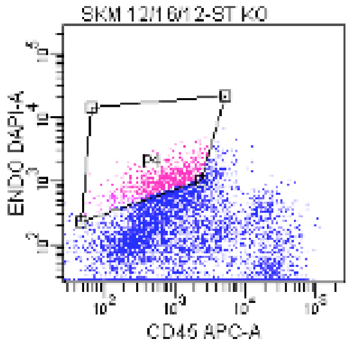

Tube: ST KO

Population	#Events	%Parent	%Total
All Events	50,000	####	100.0
SINGLETS	13,608	27.2	27.2
P1	10,062	73.9	20.1
P3	7,751	77.0	15.5
P4	717	9.3	1.4
P5	647	90.2	1.3
CELLS	12,026	88.4	24.1
CD45-PI-	5,213	43.3	10.4
ENDO+	481	9.2	1.0

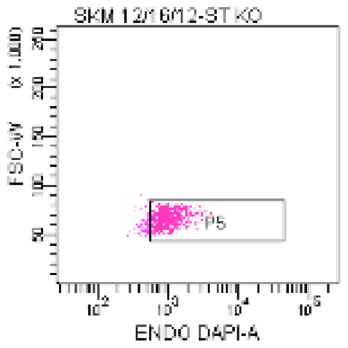

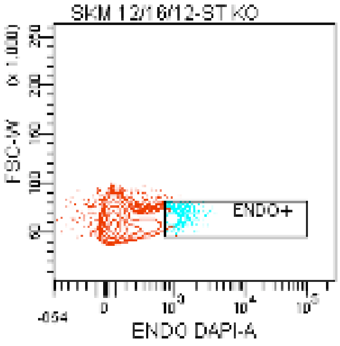

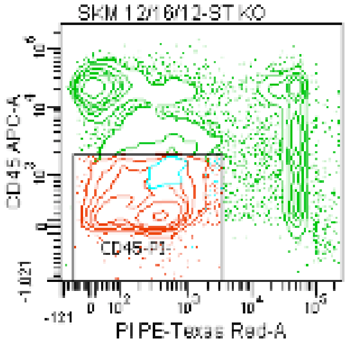

B) Aorta

PI only aorta sample

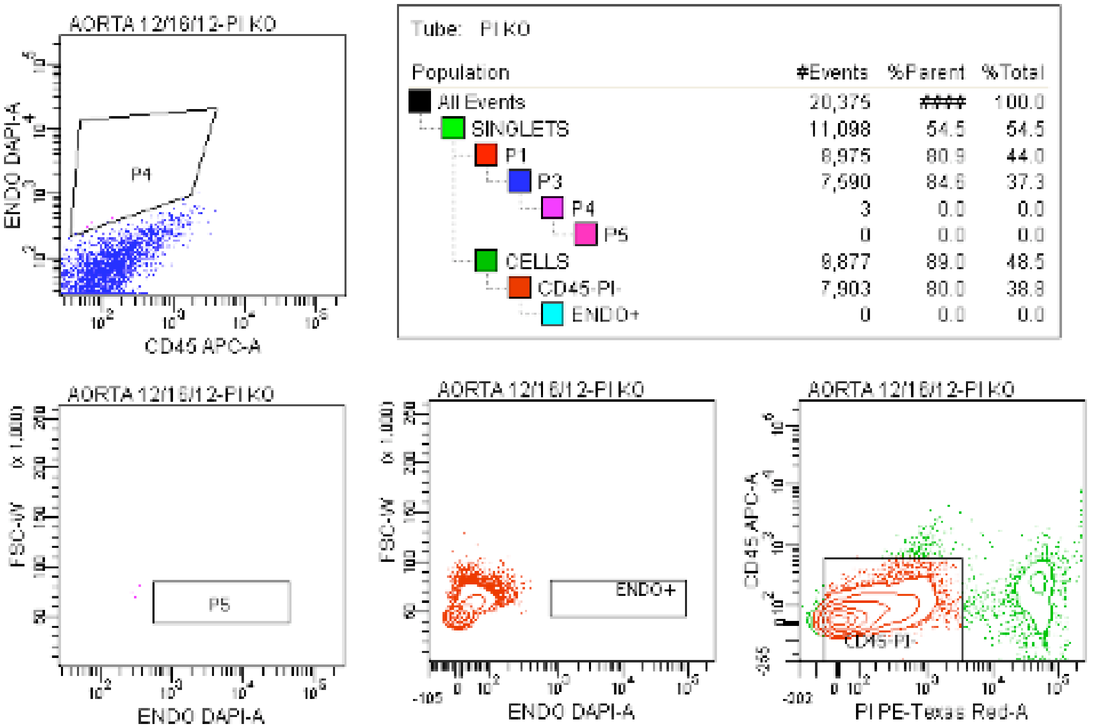

Population	#Events	%Parent	%Total
All Events	20,375	####	100.0
SINGLETS	11,098	54.5	54.5
P1	8,975	80.9	44.0
P3	7,590	84.6	37.3
P4	3	0.0	0.0
P5	0	0.0	0.0
CELLS	9,877	89.0	48.5
CD45-PI-	7,903	80.0	38.8
ENDO+	0	0.0	0.0

Aorta sample stained for Endoglin, CD45, & PI

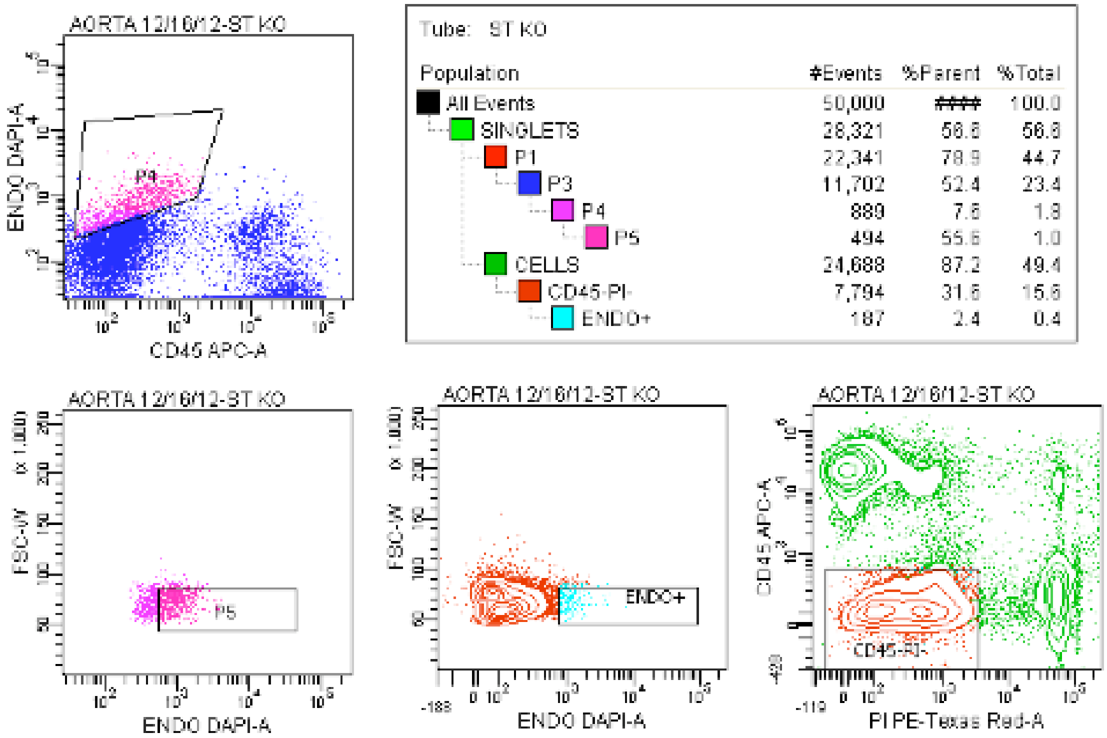

Population	#Events	%Parent	%Total
All Events	50,000	####	100.0
SINGLETS	28,321	56.6	56.6
P1	22,341	78.9	44.7
P3	11,702	52.4	23.4
P4	889	7.6	1.8
P5	494	55.6	1.0
CELLS	24,688	87.2	49.4
CD45-PI-	7,794	31.6	15.6
ENDO+	187	2.4	0.4

Additional File 4: Representative FACS profiles of the sorted endothelial population derived from the skeletal muscles and aortae of Galectin-3(-/-) and C57BL/6 mice. Collagenolytic digests of leg muscle (A) and aortae (B) of Galectin-3(-/-) and C57BL/6 mice were labeled with eFluor450-Endoglin, PE-CD45, and propidium iodide (PI) live/dead stain and sorted on a FACSAria. Endoglin, CD45, and PI signals were detected using the DAPI, APC, and PE-Texas Red channels, respectively. Dead cells that stained positive for PI and CD45+ monocytes were excluded by gating. The endoglin+ gate (bottom) was established based on unstained and PI-only negative control samples (top). Live, Endoglin+/CD45- cells were sorted directly into TRIzol reagent for subsequent gene expression analysis. Cell suspensions stained with PI alone show 0% endoglin staining, while 2.4% and 9.2% of live, CD45- cells are endoglin+ endothelial cells in the aortic and skeletal muscle digests, respectively.
