# Supplementary material for: Galectin-3 deficiency exacerbates hyperglycemia and the endothelial response to diabetes
Source: Cardiovasc Diabetol. 2015 Jun 6;14:73. doi: 10.1186/s12933-015-0230-3 (PMC4499178; doi:10.1186/s12933-015-0230-3)
Supplement: Additional file 5: — Specificity of endoglin antibody for binding endothelial cells in a skeletal muscle digest compared to the lack of non-specific binding in a cultured muscle cell suspension. [file 12933_2015_230_MOESM5_ESM.pdf]

A) Skeletal Muscle Tissue Digest

FMO

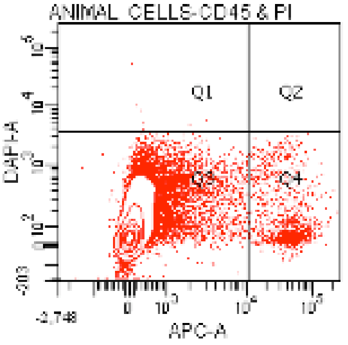

Tube: CD45 & PI			
Population	#Events	%Parent	%Total
All Events	50,000	####	100.0
P1	44,178	88.4	88.4
Q1	6	0.0	0.0
Q2	0	0.0	0.0
Q3	42,725	96.7	85.4
Q4	1,447	3.3	2.9

Stained for Endoglin

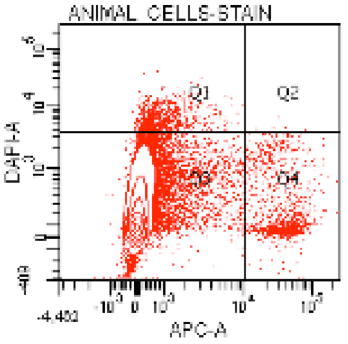

Tube: STAIN			
Population	#Events	%Parent	%Total
All Events	50,000	####	100.0
P1	43,469	86.9	86.9
Q1	818	1.9	1.6
Q2	8	0.0	0.0
Q3	41,310	95.0	82.6
Q4	1,333	3.1	2.7

B) Cultured Muscle Cells

FMO

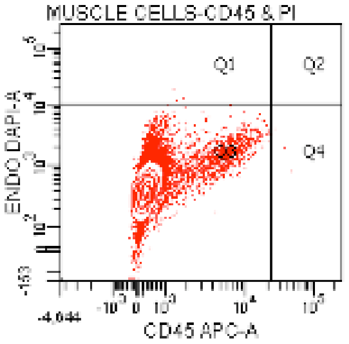

Tube: CD45 & PI			
Population	#Events	%Parent	%Total
All Events	50,000	####	100.0
P1	36,819	73.6	73.6
Q1	3	0.0	0.0
Q2	0	0.0	0.0
Q3	36,811	100.0	73.6
Q4	5	0.0	0.0

Stained for Endoglin

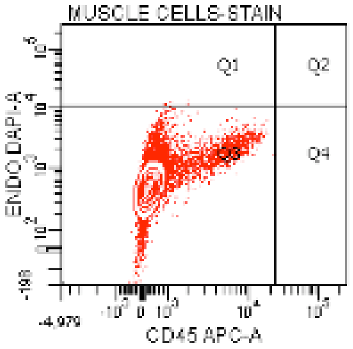

Tube: STAIN			
Population	#Events	%Parent	%Total
All Events	50,000	####	100.0
P1	36,608	73.2	73.2
Q1	6	0.0	0.0
Q2	0	0.0	0.0
Q3	36,601	100.0	73.2
Q4	1	0.0	0.0

Additional File 5: Specificity of endoglin antibody for endothelial cells in skeletal muscle digest compared to the lack of non-specific binding in a cultured muscle cell suspension. While cell suspensions of collagenolytically digested skeletal muscle tissue from mice (A) show 1.9% of live cells staining for endoglin, 0% of C2C12 muscle cell suspensions (B) stain for endoglin demonstrating that non-specific binding of our antibody is not occurring. Fluorescence minus one (FMO) samples shown on the left lack endoglin antibody but are stained for CD45 and PI uptake. Endoglin+/CD45- cells are shown in Quadrant 1; Endoglin+/CD45+ cells are shown in Quadrant 2; CD45-/Endoglin- cells are shown in Quadrant 3; CD45+/Endoglin- cells are shown in Quadrant 4.
