# Supplementary material for: Galectin-3 deficiency exacerbates hyperglycemia and the endothelial response to diabetes
Source: Cardiovasc Diabetol. 2015 Jun 6;14:73. doi: 10.1186/s12933-015-0230-3 (PMC4499178; doi:10.1186/s12933-015-0230-3)
Supplement: Additional file 6: — Transcripts identified by Ingenuity Pathway Analysis in the Cardiovascular Function and Development category, which was the most enriched biological function in the KO data. [file 12933_2015_230_MOESM6_ESM.pdf]

Additional File 6. Transcripts identified by Ingenuity Pathway Analysis in the Cardiovascular Function and Development category, which was the most enriched biological function in the KO data. Transcripts dysregulated >0.75log$_2$[fold change] in the aortic endothelium of KO and WT mice after 8 weeks of HFD vs. chow-fed controls were analyzed by IPA.

Cardiac Output	
WT	ADRA2A,ADIPOQ,AGT,DBP,IGF1,Klk1b1 (includes others),NRG1 (includes EG:112400),PDCD1,PNPLA2
KO	ABCC9,ADIPOQ,ADRA1D,ADRA2B,ARNTL,ARSB,ATP2A2,CACNB3,CACNA1G,COL4A3,DBP,EDNRA,ELN,EPHX2,FKBP1B,HLF,IGF1,IRS1,MRVI1,OXSR1,PRKCA,PTGER2,PTGES,PTGIR,SLC6A2,SNTA1,TBXA2R,WNK4
Migration	
WT	ADIPOQ,AGT,HMMR,IGF1,NRG1 (includes EG:112400),PF4,THBS1
KO	ADIPOQ,ARHGEF4,BMX,ELN,F3,FGFR1,IGF1,MMP2,PDGFRA,S100A4,S1PR2,SLIT2,SLIT3,TGFB3
CV Morphology	
WT	ADIPOQ,HEYL,MKL2,MYH10,NRG1 (includes EG:112400), NTRK3,PDCD1,PNPLA2,THBS1
KO	ADIPOQ,ADRA2B,ARSB,ATP2A2,B9D1,BCL2,CACNB3,CAV3,CNN1 (includes EG:1264),COL15A1, COL4A3, COL8A2, CYP2E1, EAF2,ECE2,EDNRA,ELN, EPHA3,F3,FGFR1, FKBP1B,GDF1, HEYL,IGF1,LEFTY1,MDK,MKL2,MMP2, NKX2-3,NTF3,NTRK3,PDGFRA,PLCE1,PRKCA,PRRX2,S1PR2,SGCD,SLC2A4, TBXA2R,TGFB3,THPO,TNFRSF11B
Growth and Proliferation	
WT	ADAMTS8,ADIPOQ,ADRA2A,AGT,ARNT,BIRC5,C5,CLEC1B,COL3A1, EPHB2,FOXS1,HEG1,HEYL,IGF1,JAM3,MKL2,NRG1 (includes EG:112400),PBX1,PF4,THBS1
KO	ADAMTS2,ADAMTS8,ADIPOQ,ADRA2B,ADRA2C,ANG,ARHGEF4,ARNTL,ATP2A2,BCL-2, BMX,C5,C6,COL15A1,COL1A1 (includes EG:1277), COL4A3,COL8A2,E2F1,EAF2,EDNRA,ELN,EPHB2,EPHB3,ESR1,F3, FGFR1,FOXS1,GPC1,HEYL,IGF1,INHBA,IRS1,LAMA2,LEFTY1,MDK,MKL2,MMP2,NKX2-3, PDGFRA,PRRX2,PTGER2,PTGES,PTGIR,PTPRF, S100A4,S1PR2,SLIT2,SLIT3,TBXA2R,TEAD4,TNFRSF11B
Vasculogenesis	
WT	ADAMTS8,ADIPOQ,ADRA2A,AGT,ARNT,BIRC5,EPHB2,HEG1,HEYL,IGF1,JAM3,MKL2,NRG1 (includes EG:112400),PBX1,PF4,THBS1
KO	ADAMTS2,ADAMTS8,ADIPOQ,ADRA2B,ADRA2C,ANG,ANGPTL2,ARHGEF4,ARNTL,B4GALNT1,BMX,C5,C6,COL15A1,COL1A1 (includes EG:1277),COL8A2,E2F1,EAF2,EDNRA,ELN,EPHB2,EPHB3,ESR1,FGFR1,GPC1HEYL,HYOU1,IGF1,INHBA,IRS1,LAMA2,LEFTY1,MDK,MKL2,MMP2,NKX2-3, PDGFRA,PRRX2,PTGER2,PTGES,PTGIR, S100A4,S1PR2,SLIT2,SLIT3,TBXA2R,TEAD4,THY1,TNFRSF11B
CV System Function	
WT	CACNA1G,JPH2,Klk1b1 (includes others),NRG1 (includes EG:112400),PDCD1,PNPLA2
KO	ADRA1D,CACNA1G,COL15A1,ELN,EPHA3,JPH2,KCNA1,MMP2,NTF3,PTGIR,SGCD,TBXA2R
Vasoconstriction	
WT	AGT
KO	ABCC9,ADRA1D,ADRA2B,EDNRA,PTGER2,TBXA2R
