# Supplementary material for: Galectin-3 deficiency exacerbates hyperglycemia and the endothelial response to diabetes
Source: Cardiovasc Diabetol. 2015 Jun 6;14:73. doi: 10.1186/s12933-015-0230-3 (PMC4499178; doi:10.1186/s12933-015-0230-3)
Supplement: Additional file 7: — The Coagulation Cascade is highly up-regulated in the aortic endothelium of diabetic KO mice compared to diabetic WT mice. [file 12933_2015_230_MOESM7_ESM.pdf]

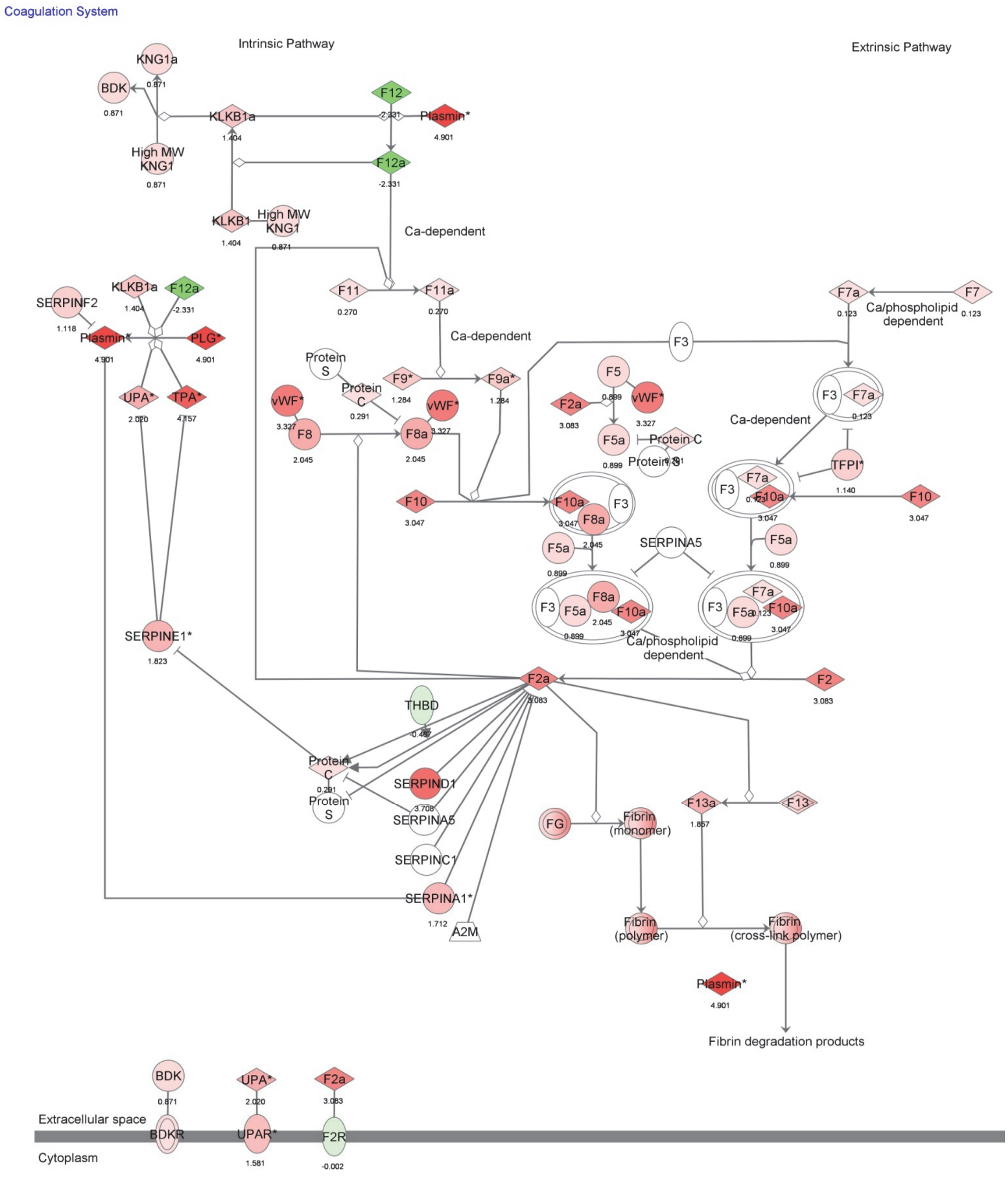

© 2000-2012 Ingenuity Systems, Inc. All rights reserved.

Additional File 7. The Coagulation Cascade is highly upregulated in the aortic endothelium of diabetic KO mice compared to diabetic WT mice. Log_2[fold change] of KO HFD/WT HFD for all aortic transcripts of one experiment were uploaded into Ingenuity Pathway Analysis. The Coagulation Cascade was found to be the most enriched canonical pathway in the aortic endothelium of knockout mice compared to WT mice. The Log_2[KO HFD/WT HFD] microarray data for the transcripts involved in this pathway are superimposed in the pathway diagram.
